# Aquatic Insect from Iran for Possible Use of Biological Control of Main Vector-Borne Disease of Malaria and Water Indicator of Contamination

**Published:** 2018-03-18

**Authors:** Zahra Saeidi, Hassan Vatandoost

**Affiliations:** 1 Department of Medical Entomology and Vector Control, School of Public Health, Tehran University of Medical Sciences, Tehran, Iran; 2 Department of Environmental Chemical Pollutants and Pesticides, Institute for Environmental Research, Tehran University of Medical Sciences, Tehran, Iran

**Keywords:** Aquatic insects, Arthropod-borne diseases, Iran, Water quality

## Abstract

Iran has a wide variety of zoogeographical regions and different seasons. Here are some important mosquito-borne diseases. Mosquitoes normally live in waters. Its aquatic insect fauna is highly unexplored. To being resolved this faunal gap, a variety of literature records from previous century in different parts of Iran was reviewed. In some southern and southeastern foci in Iran, Malaria is still a main endemic disease which is unstable with two seasonal spring and autumn peaks even though Iran is lunching Malaria elimination. This review article showed the wide variety of aquatic insects throughout the country. Researchers can discuss water pollutant and its quality by using aquatic insect fauna as well as biological control for vectors. Types of aquatic insects and macroinvertebrates sampling can be useful for water quality monitoring as indicators. Looking at aquatic insects’ life in water could be one of the most cost-effective and the easiest method to assess the water contaminations by different pollutants and will provide a guideline for scientific communities and environmental agencies for decision making.

## Introduction

There are some important arthropod-borne diseases in Iran including Malaria, Cutaneous leishmaniasis, Visceral leishmaniasis, Crimean-Congo hemorrhagic fever, tick relapsing fever, Furthermore scorpions are one of the risk factors for life in some parts, while other arthropod-related diseases such as myiasis exist more or less across the country. Some probable Arthropod-borne disease in the future may be: Q-fever, Papatasi fever, Tularemia, Rift valley fever, Dengue fever, Yellow fever, West Nile viruses, Lactodictism (spider bite), Plague, scabies, Nuisance insects of horseflies and Culicidae mosquitoes, Cockroach-borne diseases, damages by fire ants, blister beetles and bee stings.

In Iran with about 15000 annual cases of the disease in recent years, malaria is known as one of the most important parasitic infectious diseases. Locally transmitted cases have dropped to 500 recorded cases in 2013. Three most prevalence provinces in Iran are Sistan-Baluchestan, Hormozgan and Kerman which located in south and southeastern part of the country. The rifest route of transmission is immigration from Afghanistan and Pakistan to this area (Ministry of Health, annual report). You can find a considerable decline of malaria burden in Iran during last 20 years. The disease cases have been reduced from about 100000 cases in 1991 to 246 autochthonous cases in 2014. Most of the transmitted cases are reported from the south-eastern part of the country that is related to population traffic across Pakistan border beyond the difficulties in malaria control. Recent malaria number reported is 42 cases all over the country including 23 local malaria patients, 12 imported cases and seven relapsed ones before August 2016. Majority of researchers have worked on various aspects of malaria such as insecticide resistance monitoring (1–10) new records, sibling species and molecular studies (11–18). Some researchers have worked on vector control using novel methods (19–24), faunestic study (25–26), Larval control using various plants (3, 27–38), using bed nets and long lasting impregnated nets (39–46), Study on morphology (47–49), Malaria epidemiology (50–54) Malaria vector ecology (18, 39, 52, 55–60 ), Biodiversity (53, 61), Community participation (62), Vector control (63), Repellent evaluation (31, 64), susceptibility against insecticide (65–67), Anthropophilic index of malaria vectors (68–69) Training (70) is nominated as malaria training center by WHO researchers also can find several reports on different aspects of malaria vectors done in recent years (21, 71–87).

Although Iran has a vast geographical area with a wide range of diversity in climate and animal including insects, its aquatic insect’s fauna remains largely unexplored for years. With a total area about 1.65 million Km ^2^ , around 7% covered with water- Iran is one of the large countries ranked eighteenth in the world (88–89). The aquatic insect has a critical role in biomonitoring of water safety or water contamination. It is inevitable to use such kind of insect to evaluate water quality as a biological indicator and can help us as water resource management. Aquatic insects a vital role in energy flow in fresh water and they are important in food web between aquatic animals.

## Historical overview of Aquatic Insects from Iran

### Past century

Iran aquatic insects have been studied since 1965 by a hand full of researchers. Vassil Gueorguiev recorded *Methles rectus* from Iran, but he did not publish the exact location (88). Afterwards, in 1976 some researcher from another field such as environment researchers who surveyed on mayfly and stonefly to determine the acute metal toxicity of some heavy metals such as lead, copper, zinc, and silver. They were found more tolerant than most fish to heavy metals. This study indicated that aquatic insect can help us as effective biological monitors of heavy metals pollution (91). Subsequently a survey was conducted on water beetles of Southwestern Iran and reported Haliplidae (two genera, two species), Dytiscidae (16 genera, 24 species), and Gyrinidae (two genera, two species). Hydrophilidae (10 genera, 34 mostly unidentified species) (92). A researcher from a university of Shiraz focused on life history, morphology and behaviour of the immature stages of a coleopteran, Hydrophilidae in laboratory condition (93). After about 20 years of water beetle collecting from a wide range of area, habitat and provinces in Iran founded a small number of *M. rectus* sharp in a few places in Guilan Province in a collection made in 1976, 1993 and 1995 in Southern part of Caspian Sea, northern Iran (94).

### Current century

During 2000–2002 a study on aquatic beetle of Tabriz region, East Azarbaijan, Northwestern Iran was conducted and four species out of five species of the family Hydraenidae reported a new record (95). During 2001–2005 some specimen collected by Vafaei et al. (96) in Markazi Province central Iran and they established the presence of 24 species of aquatic beetles (Coleoptera: Polyphaga) belonging to 13 genera and five families. In other publication, they claim that they found 33 species of diving beetles belonging to 18 genera during same time and same places (97). In 2005 another team worked on a descriptive study of aquatic insects’ fauna in Kashan, central Iran. During nine rounds of sampling from four maturation artificial ponds they reported as followed: Diptera order (52%), including Chironomidae and Culicidae families, Hemiptera (24%) Corixidae, Notonectidae, Copepodae and Copepodidae families, Ciclopodidae (12%), Hydroacarina (9.5%), Coleptera (0.77%), Aranida (0.67%), Hymenoptera (0.58%), Odonata (0.48%) (98). In another publication, 31 different Plecoptera reported from different families and two families of Ephemeroptera (99).

During 2006 and 2007 39 species have been found belonging to 16 families in Zanjanrud, Zanjan Province. Three specimens belong to Lygaeidae, Scutelleridae and Reduviidae were identified at the genus level. Among them, there are some predators’ species such as *Anthocoris nemorum*, *Nabis pseudoferus*, *Notonecta viridis*, *Velia affinis*, *Gerris maculates*, *Hydrometra stagnorum*. The most frequent Species belonged to Pentatomidae. All species were first records from the Zanjan Province (100).

Some families of Coleoptera such as Dytiscidae, Gyrinidae, Helophoridae and Hydrophilidae with a new record and notes on the rare species *Coleostoma transcaspicum* Reitter, 1906 from North part of Tehran Province was reported (101). Work on Odonata as effective predators in the rice field and other sites in Mazandaran Province North of Iran (2003–2006). They found 30 species from 19 genera and eight families of Odonata (Anisoptera and Zygoptera suborder. In Anisoptera sub-order, Aeshnidae, five species, one species of Corduliidae, two species of Gomphidae, 13 species of Libellulidae. In Zygoptera suborder, one species of Calopterygidae, six species of Coenagrionidae, one species of Euphaeidae, one species of Platycnemididae (102). A survey in Zayande Rud River in Esfahan Province, central Iran during one year and in eight stations who found that the water quality can have an effective impact on diversity and richness of benthic macroinvertebrate (103) recorded total of 47 species belonging to17 Families of Heteroptera in Ghara Dagh forest, East Azarbayjan, Northwestern Iran: Among them, the species *Nabis pseudoferus*, *Notonecta viridis*, *Anthocoris nemorum*, *Velia affinis*, *Hydrometra stagnorum* and *Gerris maculates* were predators. The most abundant species belonging to Pentatomidae. They reported 32 species as new records for the studied area. Newly introduced species, *Stinctopleurus crassicornis* and *Stinctopleurus punctatonervosus*, registered for Iran insect fauna (104).

In another study that carried out in East Azarbaijan Province on Heteroptera, they found 28 species from 12 families and *Polymerus brevirostris* Knight, 1925 was a new record (105). In Ardabil East Azarbaijan Province Northwestern Iran. Two species of aquatic beetles belonging to two genera that did not study before from two sampling site during 2000–2008. They faced *Peltodytes* Regimbart, 1878 and *Haliplus* Latreille, 1802 (106). A species *Haliplus heydeni* Wehncke, 1875 was a new record of Iran. In another teamwork in Neka County, the Mazandaran Province, Northern Iran five species of four genera were found in tree holes during 2009. They reported *Anopheles plumbeus*, *Culisetaannulata*, *Culex pipiens*, and *Ochlerotatus geniculatus* by larval collection, *Ochlerotatus pulcritarsis*by adult collection and *Oc.geniculatus*, 55.87%, *Ochlerotatus echinus* 1.33%, Oc. *pulcritarsis* 8.8%, *Cx. pipiens* 33.8%, and *An. plumbeus* 0.2% of bait net collection. They found some *Cs. Annulata* larvae in low abundance in cavities of trees for the first time (61). During 2008– 2009 İncekara et al. (107) collected 42 species of aquatic beetle (Coleoptera: Hydrophiloidea) belonging to 13 genera and three families (Helophoridae, Hydrochidae and Hydrophilidae) in Tehran, Mazandaran, Guilan, Qazvin, and Sanandaj in Kordestan Provinces in Iran. They reported 11 new species from Iran in this survey. During 2009–2011 on the work on the aquatic insect of Karun River, Ahvaz, Khuzestan Province, Southwestern Iran revealed Damselflies and Dragonflies nymphs of Odonata order, five genera from four families and all of them were the new records from this area.

### Recent years

**Fig. 1.**
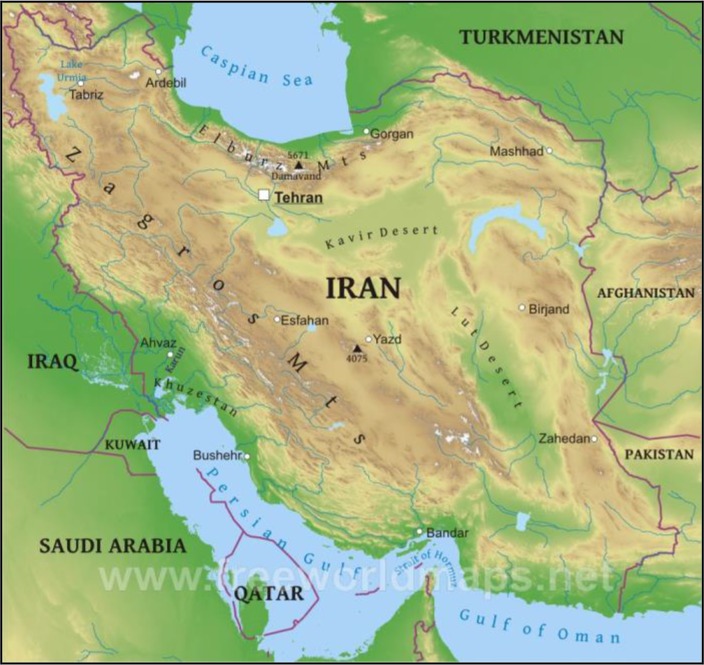
Map of Iran showing some main rivers and natural events

These days we are able to find some more articles about Iran aquatic insects that worked in a wide range of natural areas (Fig. 1). It seems more researchers know the importance of aquatic insects as biological control and water indicator and they are interested in investigating on them. In 2011 Salavatian et al. who worked on feeding behavior of Brown trout, *Salmo trutta fario*, published a paper that shows us this fish fed on 32 animal groups including some insects such as Chironomidae (88.6%), Simulidae (60%), Baetidae (51.4%) and Tipulidae (50%) that they were most frequent food in its gut. They showed that the proportion of consumed food by Brown trout was Diptera 91.5% (Chironomidae pupa and larvae 85.8%), Coleoptera 6.4% and others 2.1% (109). Other team surveyed aquatic insects’ fauna of Karun River, Ahvaz City, Khuzestan Province, Southwest of Iran. They reported 57 species belonging to seven orders and 22 families, Collembola (1 species), Ephemeroptera (4 species), Odonata (6 species), Hemiptera (9 species), Coleoptera (34 species), Diptera (2 species) and Trichoptera (1 species). The most abundant species in this study was the beetle *Hydroglyphus signatellus* Klug, 1834 (Coleoptera: Dytiscidae) (110). An Ecological Risk Assessment (ERA) for Shadegan wetland, Khuzestan Province, Southwest of Iran to assess the risk to zoo-plankton, phytoplankton, invertebrate, insect larvae, and fish affected by Five pesticides, DDT, Aldrin, Dieldrin, Lindane and Ametryn. Insect larvae (*Chironomus* sp) like other creature are highly at risk of harmful pesticide were conducted (111). Study on Tajan river macroin-vertebrate communities’ distribution in Mazandaran Province, Northern Iran and south part of Caspian Sea. They realized that the dissolved oxygen, turbidity, water temperature, pH and TSS were the most critical physicochemical factors to affect the distribution of them (112).

Work on the potential aquatic habitats for *Anopheles* larvae from Indian Remote Sensing Satellite (IRS) image and digital elevation model of the area using GIS by monthly sampling from Surface water bodies during 2009– 10 for anopheline larvae carried out. The lowest and highest frequencies were in February and April, respectively. *Anopheles culicifacies* was Dominant species (53). In a study conducted in Gahar Lake, Lorestan Province in three different seasons they found most and least variety and abundance in spring and autumn respectively. Maximum density belonged to Simulidae and Chironomidae (113).

Ghahari collected, identified and published about 19 species from nine genera (Micronecta, Corixa, Sigara, Aquarius, Gerris, Hydrometra, Anisops, Chartoscirta, Saldula) of aquatic and semiaquatic Heteroptera from the families Corixidae, Gerridae, Hydrometridae, Notonectidae, Saldidae from southern areas of Caspian Sea, Northern Iran (114). From 16 different sites in Iran, 23 nominal species are now identified, including some new records for *Simulium crassicaulum* (Rubtsov) and *Simulium alajense* Rubtsov, and the southernmost world record for *Simulium transcaspicum* Enderlein in Iran. Multiple cytoforms of the *Simulium aureum* group, *Simulium bezzii* complex, and *Simulium ornatum* group were found (115).

Shaverdo et al. reported 21 species of diving beetles Dytiscidae from Ahvaz, Khuzestan Province, southwest Iran. *Cybister lateralim* arginalisponticus, 1882, Hydroporus inscitus 1882, and *Laccophilus sordidus* 1882 are reported from Iran for the first time (116). Shayeghi et al. reported a variety of aquatic orders, two families of Hemiptera (Gerridae and Notonectidae) Odonata (Coenagrionidae), Coleoptera (Carabidae), and pro stigmata from the family of Hydrachindae in Zayanderood, Esfahan Province, Central Iran during 2011 (117). In another study in the same area and the same year, they collected 741 specimens of aquatic insects including seven families and 12 genera of two orders. The order of Diptera (92.31%) including Culicidae, Syrphidae and Chironomidae and Coleoptera (7.69%) including Gyrinidae, Dytiscidae, Haliplidae, Hydrophilidae families (76).

Maleki-Ravasan et al. (118) conducted a bi-seasonal study in Lavasan River, Northeastern Tehran, the most abundant species between 14 families and 62 Trichoptera species belonged to the Hydropsychidae. They reported the presence of Annulipalpian *Hydropsyche sciligra* H Malicky, 1977 in that district. Habitat water quality of this species reported resemble human drinking water and presence of *Physa acuta* (snail) and *Capoeta buhsei* (fish) in the sampling area indicated inferior quality. Darilmaz et al. listed 27 species and subspecies of 17 genera of the families Dytiscidae, Haliplidae, Noteridae and Gyrinidae (Coleoptera: Adephaga) from Alborz, Gilan, Mazandaran, Qazvin, and Tehran Provinces northern Iran (119). A total of 9 families in Shapoor River in Bushehr region during 2012 (120).

Researchers evaluated aquatic insects’ fauna in Golestan Province, North of Iran in different sites during 2011–2012. They published different stages of Diptera 64.54% (Culicidae, Chironomidae, Tabanidae, Simulidae, Sciomyzidae families), Heteroptera 11.03%, Ephemeroptera 9.53% (Heptagenidae, Baetidae), Trichoptera 7.07% (Limnephilidae), Odonata 4.82% (Aeshnidae, Gomphidae, Libellulidae) and Coleoptera 2.99% (Dytiscidae, Gyrinidae) in this study. They reported some water surface insects such as Gerridae, Corixidae, Hydrometridae, Nepidae families (121). A study in Karaj River, North of Iran. 211 samples of three orders; Plecoptera, Trichoptera and Ephemeroptera and seven genera (Perla, Isoperla, Hydropsyche, Cheumatopsyche, Baetis, Heptagenia and Maccafferium) from five families (Perlidae, Perlodidae, Hydropsychidae, Batidae, Heptagenidae) were found. Order of Plecoptera was the most predominant order then Trichoptera (122). Investigated bio-diversity of culicid mosquitoes from Keka revealed 5270 specimens belonging to four genera and 14 species in Northern Iran. They reported one dominant species, two dominant species, two subdominant species, two rare species and eight sub rare species by using Heydemann classification (123). Southwest of Iran, Bashagard district is one of the most important areas because of Malaria transmission collected research conducted revealed totally 5150 larvae from 36 different larval habitats. They recorded six species: *An. culicifacies* (29.36%), *An. moghulensis* (25.20%), *An. dthali* (18.02%), *An. superpictus* (17.24%), *An.turkhudi* (5.17%) and *An. stephensi* (5.01%). They investigated water quality and they stated abundant Anophelin larvae existed in permanent and full sunlight habitat with no vegetation and algae. Larval density had the correlation with water temperature. Some factors also had the specific impact on larval abundance and distribution such as conductivity, total alkalinity, chloride and sulphate. Knowing of this data and correlation between them can be considered for sufficient planning and implementing Malaria elimination program (124). In Bashagard area epidemiological and entomological aspects to determine malaria situation, species composition of anopheline mosquitoes and susceptibility status of main vectors to insecticides/ larvicides during 2002–2010 were conducted. They have reported *An. culicifacies*, *An. dthali*, *An. stephensi*, *An. superpictus*, *An. fluviatilis*, *An. moghulensis*, *An. turkhudi* and *An. apoci* with two peak in April and October. They have found resistance against DDT in *An. stephensi* and tolerance against Deltamethrin and Bendiocarb.Their larvae found susceptible against all larvicides except for *An. stephensi* with tolerance against Fenthion (125). In summer 2014 Shayeghi et al. carried out a study in Sabalan mountainous river, in different sites around Meshginshahr, Ardabil Province, Northwestern Iran. They reported six orders (Coleoptera, Ephemeroptera, Hemiptera, Diptera, Plecoptera and Trichoptera) including 12 families (Helmidae, Leptophlebiidae, Ecdyonuridae, Corixidae, Culicidae, Simuliidae, Perlidae, Leptoceridae, Hydropsychidae, Chironomidae, Caenidae and Baetidae) among 262 specimens. They wrote that most abundant families were Culicidae (61.55%) and a few number of Plecoptera: Perlodidae (0.5%) (126).

## Conclusion

This review article will provide a clue for management of vector control as well as indicators for water classification.
